# Application of kDNA Minicircle PCR-RFLP to Characterize *Leishmania donovani* Clinical Isolates Obtained from Post-Kala-Azar Dermal Leishmaniasis in Eastern Nepal

**DOI:** 10.1155/2019/9392414

**Published:** 2019-07-30

**Authors:** Ojesh Pokhrel, Keshav Rai, Narayan Raj Bhattarai, Suman Rijal, Arpana Rijal, Basudha Khanal

**Affiliations:** ^1^Department of Microbiology, B. P. Koirala Institute of Health Sciences, Dharan, Nepal; ^2^Department of Internal Medicine, B. P. Koirala Institute of Health Sciences, Dharan, Nepal; ^3^Department of Dermatology, B. P. Koirala Institute of Health Sciences, Dharan, Nepal

## Abstract

Post-kala-azar dermal leishmaniasis (PKDL) is a skin manifestation of visceral leishmaniasis (VL) which develops after apparent cure in some patients. PKDL is considered as the potential reservoir for the VL infection. Molecular epidemiological characterization of *L. donovani* isolates obtained from VL and PKDL isolates is essentially required in order to understand the transmission dynamics of the VL infection. To date, genetic variation among the VL and PKDL *L. donovani* isolates was not fully elucidated. Therefore, 14 clinical isolates from VL and 4 clinical isolates from PKDL were speciated by *hsp70* and rDNA genes. Further characterization of *L. donovani* by *haspB* PCR demonstrates two different genotypes. All PKDL isolates have the same genetic structure. kDNA PCR-RFLP assay revealed 18 different genotypes; however, structural analysis showed the two distinct kDNA genotype population (*k* = 2). The kDNA fingerprint patterns of parasites from hilly districts were clustered separately from low-land districts. Therefore, further study with a large number of samples is urgently required for systematic characterization of the clinical isolates to track the molecular epidemiology of the *Leishmania donovani* causing VL and the role of PKDL as a reservoir.

## 1. Introduction

Post-kala-azar dermal leishmaniasis (PKDL) is a progression of visceral leishmaniasis (VL) that demonstrates lesions or hypopigmented skin rashes in patients even after successful treatment of VL [1]. This cryptic PKDL is characterised by papular, macular, and/or nodular lesions throughout the body, mainly demonstrated on the face, trunk, legs, arms, and genitals. About 5–10% of patients infected with VL develop PKDL in the Indian subcontinent [2]. In Nepal, PKDL occurs in 2.4% of previously treated cases of VL with 1.4% estimated to be at risk within 2 years of treatment [3] whereas in case of India, the interval ranges between 2 and 7 years [4].

The PKDL skin lesion has a tendency to become chronic and harbours the parasite, hence considered as a reservoir, especially challenging the elimination programme of VL from the Indian subcontinent [5, 6]. The clinical, epidemiological, parasitological, and immunological development of PKDL is not yet fully understood [7]. In fact, the eradication of PKDL could be an essential factor for the current VL elimination programme. Moreover, the asymptomatic VL infection is also considered as the reservoir for VL transmission that threatens for VL elimination in the Indian subcontinent [8]. The immunological responses are also found to be different in patients with VL and PKDL [2]. Hence, the molecular epidemiology of *L. donovani* causing different clinical manifestations is required to understand in order to minimize the risk of VL elimination in the Indian subcontinent. The genetic analysis has shown the significant heterogeneity among the Indian *L. donovani* isolates that cause different clinical presentations such as VL and PKDL [9, 10]. But no such data on genetic characterization of *L. donovani* isolates from Nepal were available yet. Therefore, we focused on the study of the genetic characterization of VL and PKDL parasite isolates from Nepal using available highly efficient molecular assay kDNA minicircles PCR-RFLP together with *haspB* PCR assay.

## 2. Materials and Methods

### 2.1. Sample Collection and Parasite Isolation

The sample collection consisted of *L. donovani* clinical isolates from 14 VL and 4 PKDL patients who were presented between January 2013 and June 2014 at B. P. Koirala Institute of Health Sciences (BPKIHS), a tertiary care hospital in Dharan, Nepal. Ethical clearance was obtained from Institutional Ethical Review Board (IERB), BPKIHS, Nepal. Written consent was obtained from all patients or from parents or guardians in case of children. Promastigote forms of *L. donovani* were isolated from bone marrow aspirates of VL patients and skin punch biopsy of PKDL patients. Parasite culture was performed by inoculating the clinical specimens in Tobie's blood agar medium with Locke's overlay, with 200 IU/ml penicillin and 200 *μ*g/ml streptomycin [11]. Once the parasites were fully grown from the clinical material, they were transferred to M199 (Sigma-Aldrich, cat. no. 2520) with 20% fetal calf serum (Invitrogen, cat. no. 10270) + adenosine + haemin.

### 2.2. Parasite DNA Extraction and Species Identification

DNA was extracted from parasite cultures using the QIAamp DNA mini kit (Qiagen, http://www.qiagen.com). Parasites in late logarithmic growth phase were washed thrice with sterile PBS solution, and DNA was eluted in 200 *μ*L AE buffer. DNA concentration and purity were verified by spectrophotometric measurement with the BioPhotometer plus (Eppendorf). The *Leishmania donovani* species was confirmed using PCR-RFLP analysis of the heat-shock protein 70 gene (*hsp70*). The fragments referred to as HSP70-N [12, 13] were digested with restriction enzymes HincII [14] (Promega, cat. no. R6031) and MluI (Promega, cat. no. R6381).

In addition, the *Leishmania* genus screening was also done by rRNA-specific PCR with the forward primer 18S-L-F (5′-CGTAGTTGAACTGTGGGCTGTGC-3′) and the reverse primer 18S-L-R (5′-ACTCCCGTGTTTCTTGTTTCTTTGAA-3′) [15]. We followed the methods described by Ostyn et al. [16]. The PCR were done in 25 *µ*l containing 1X PCR buffer, 2.5 mM MgCl_2_, 200 *µ*M of each dNTP (Eurogentec), 0.1 mg/ml of bovine serum albumin (Promega), 0.8 *µ*M of each primer (Sigma-Aldrich), and 0.5 unit of HotStar Taq DNA polymerase (Qiagen, cat. no. 203605). Finally, 100 pg of template DNA was added. The thermocycling program was (i) initial denaturation at 94°C for 5 minutes; (ii) 40 cycles of denaturation at 94°C for 30 seconds and 60°C for 30 seconds and extension at 72°C for 45 seconds; and (iii) final extension at 72°C for 5 minutes. In addition, *L. donovani* isolate DNA (BPK282/0 cl4) as a positive control and two no-template controls were included in each experiment. The amplified PCR products were visualised on 2% agarose gel after electrophoresis at 5 V/cm and ethidium bromide staining. Hence, the positive PCR results show DNA band at 115 bp. In order to rule out the false-negative PCR results, plasmid DNA cloned with oligonucleotide (236 bp) was used as an internal control [17].

### 2.3. *L. donovani* Genotyping Assay

#### 2.3.1. *haspB* PCR

The *haspB* PCR for K26 antigen detection was used as species screening for *L. donovani* by primers K26f (5′-ACGAAGGACTCCGCAAAG-3′) and K26r (5′-TTCCCATCGTTTTGCTG-3′) [18]. The PCR master mix was prepared in 50 *µ*l containing 1X Qiagen PCR buffer, 1.5 mM MgCl_2_, 200 *µ*m of each dNTP (Eurogentec), 0.5 *µ*M of each primer (Sigma-Aldrich), and 1 U of HotStar Taq polymerase. Amplification was done with (i) initial denaturation step of 95°C for 5 minutes; (ii) 35 cycles of denaturation at 94°C for 30 seconds, annealing at 50°C for 30 seconds, and extension at 72°C for 60 seconds; and (iii) a final extension at 72°C for 10 minutes. Out of two *haspB* genotypes identified, genotype A has amplicon size of 640 bp and genotype B has amplicon size of 320 bp.

#### 2.3.2. kDNA PCR-RFLP

Genotyping of *L. donovani* clinical isolates was also done by kDNA PCR-RFLP assay. First, the kDNA minicircle is amplified by PCR using the primer pairs BPKMINFOR (5′-CTGGGGGTTGGTGTAAAATAGGGC-3′) and BPKDNAMINREV (5′-CCCGATTTTTGGCATTTTTGG-3′). The PCR master mix was prepared in 50 *µ*l containing 1X PCR buffer, 2 mM MgCl_2_, 200 *µ*M of each dNTP (Eurogentec), 0.5 *µ*M of each primer (Sigma-Aldrich), 1 U of HotStar Taq polymerase, and about 1 ng of template DNA. Amplification was done with (i) initial denaturation step of 95°C for 5 minutes; (ii) 40 cycles of denaturation at 94°C for 1 minute, annealing at 60°C for 1 minute, and extension at 72°C for 1 minute; and (iii) a final extension at 72°C for 10 minutes. The PCR amplicon verified at 800 bp was precipitated with 0.3 M sodium acetate and 100% ethanol at −20°C overnight. The pellets were washed with 70% ethanol, and restriction digestion was done on precipitated PCR amplicons. Restriction digestion was done in a total of 20 *µ*l of the buffer with 10 units of HaeIII and incubated at 37°C overnight. The reaction was stopped by heating to 80°C for 20 minutes, and then the fragments were analysed by electrophoresis in 3.75 V/cm in a 3% metaphor agarose gel (Lonza, USA), after staining with ethidium bromide.

The patterns of fingerprint bands were analysed using Gel Compare II 6.6 (Applied Maths, Ghent, Belgium) software. The spectral analysis was used to remove the background noise using the gel picture without signal saturation. Other gel artefacts were filtered out using the DNA ladder at both sides and middle part of each gel. Only the region between 200 and 600 bp was considered for fingerprint analysis, following the digested fragments demonstrated by Bhattarai et al. Similarity matrix was made and UPGMA dendrogram was built. The validity of each branch was evaluated using Pearson's correlation coefficient between the dendrogram derived and the original curve similarities. To assure the reliability of the clusters, the duplicate experiments were done. Furthermore, four consecutive subcultures of two parasites 63291-Fx and BPK-PKN468 were examined to minimize the experimental variations.

The Bayesian clustering approach was also employed to estimate the kDNA population structure using Structure v2.3.4 [19]. *L*(*K*), *L*(*K*)′, and *ΔK* were computed from 50 runs for 1 ≤ *K* ≤ 10 using a burn-in of 10^4^ and run length of 10^5^ iterations. For each RFLP band, allelic diversity for each population was calculated for the number of individuals.

## 3. Result

**Figure 1:**
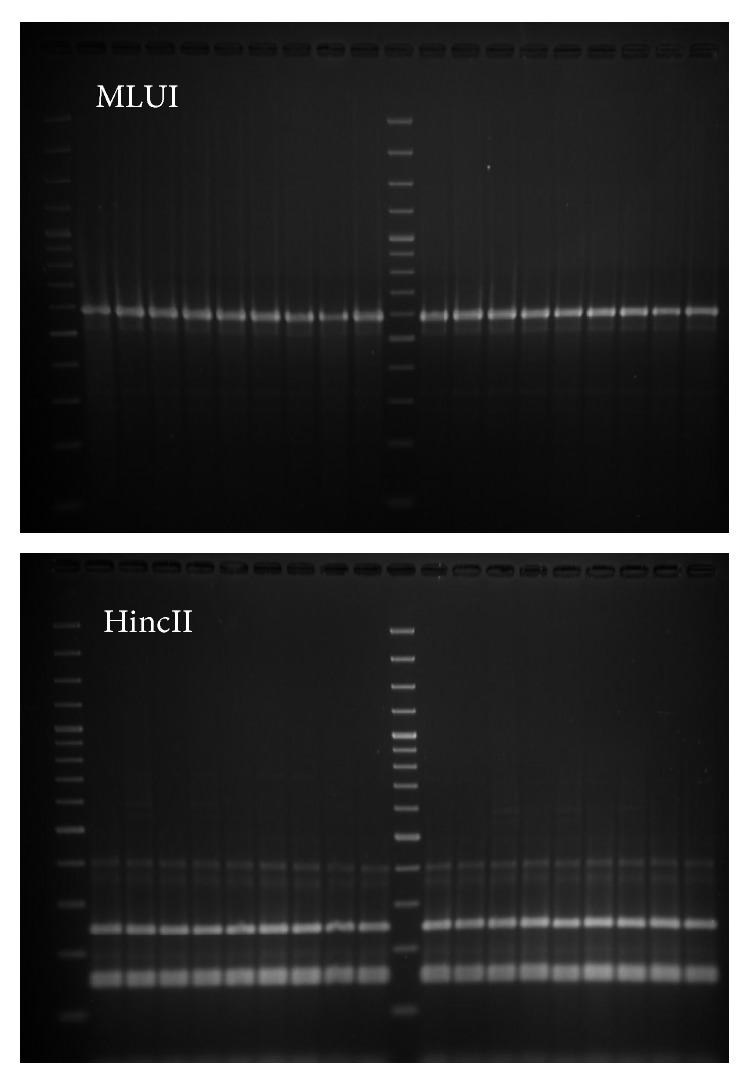
Gel picture of *hsp70* restriction digested products showing the isolates of *L. donovani* species.

**Figure 2:**
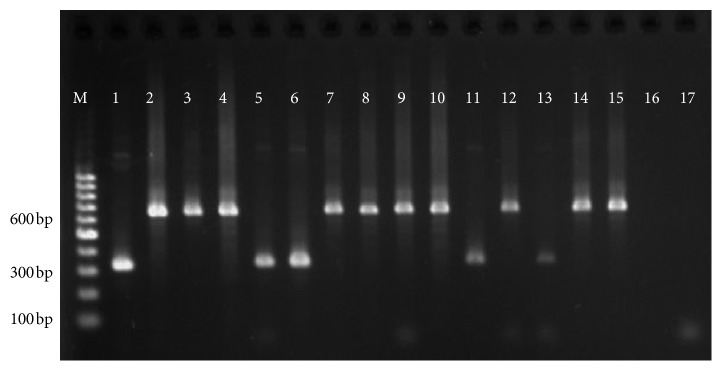
Gel picture of *haspB* PCR showing PCR products at 320 bp and 640 bp. Note: M: DNA ladder, 1: BPK806/0, 2: BPK811/0, 3: BPK814/0, 4: BPK813/0, 5: 63232-Fx, 6: 63233-Fx, 7: 63238-Fx, 8: 63241-Fx, 9: 63245-Fx, 10: 63256-Fx, 11: 63269-Fx, 12: BPK-PKN435, 13: 63277-Fx, 14: BPK-PKN467, 15: BPK-PKN468, 16: negative control N1, and 17: negative control N2.

**Figure 3:**
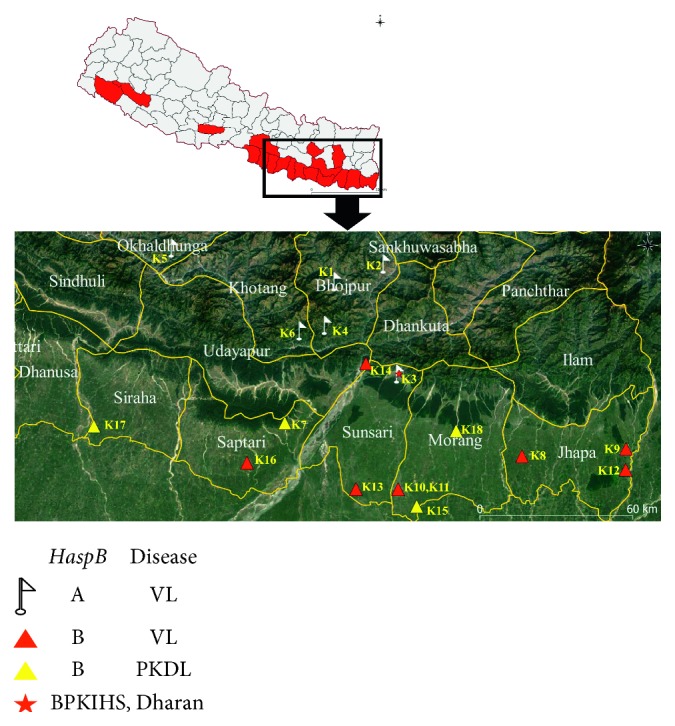
Spatial distribution of different genotypes and disease in Eastern Nepal.

All parasite isolates were confirmed as *L. donovani* species by *hsp70* as shown in Figure 1 and rRNA assay. *haspB assay* was classified into group A (*n* = 6) and group B (*n* = 12) as shown in Figure 2. Most of the group A parasites were originated from hilly districts, namely, Bhojpur, Khotang, and Okhaldhunga, except the one parasite from Sunsari (Figure 3). In contrast, the group B parasites were isolated from low-land districts (Terai) such as Jhapa, Morang, Sunsari, Saptari, and Siraha. In case of clinical presentation in patients, group A parasites were isolated only from VL patients whereas group B parasites were isolated from both VL and PKDL patients as shown in Table 1.

**Figure 4:**
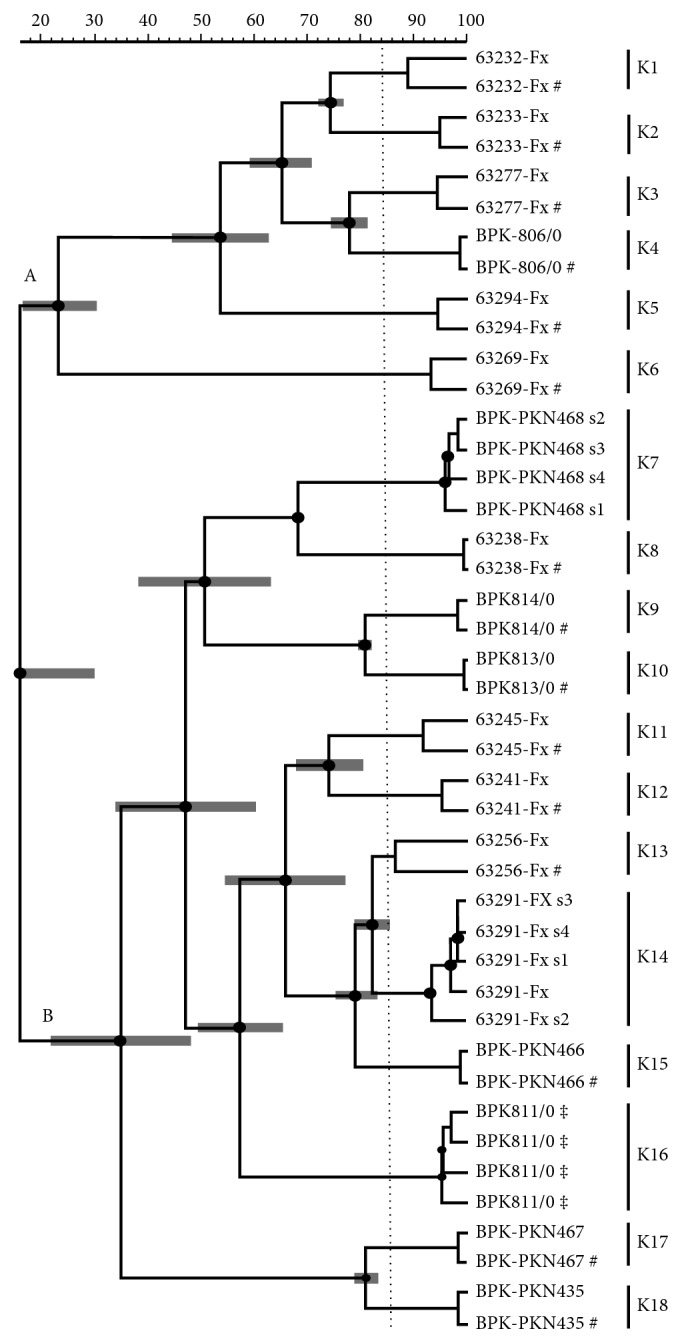
UPGMA dendrogram of all the 18 isolates obtained from kDNA PCR-RFLP (HaeIII) fingerprint data considering the densitometric curve. #: duplicate samples; s2–s4: four consecutive subcultures; ‡ control samples. A and B: groups separated according to *haspB* PCR product size.

**Figure 5:**
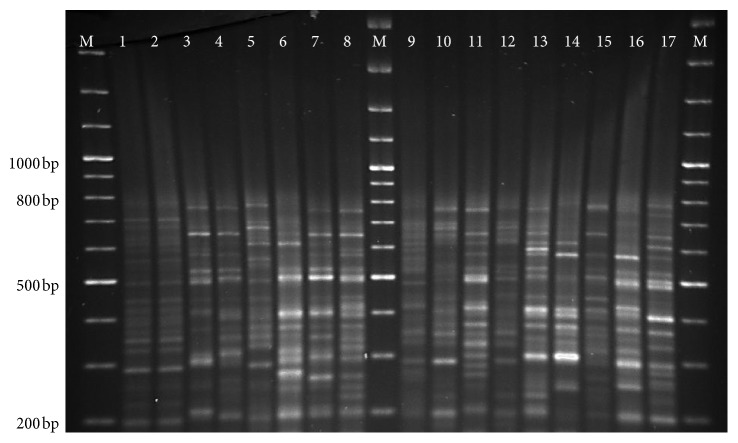
Gel picture of kDNA PCR-RFLP using HaeIII restriction digested products. Note: M: DNA ladder, Lane 1: BPK806, Lane 2: BPK806 (duplicate sample), Lane 3: BPK813, Lane 4: BPK814, Lane 5: 63233-Fx, Lane 6: 63241-Fx, Lane 7: 63245-Fx, Lane 8: 63256-Fx, Lane 9: 63269-Fx, Lane 10: 63277-Fx, Lane 11: 63291-Fx, Lane 12: 63294-Fx, Lane 13: BPK-PKN435, Lane 14: BPK-PKN467, Lane 15: BPK-PKN468, Lane 16: BPK811, and Lane 17: BPK-PKN466.

**Figure 6:**
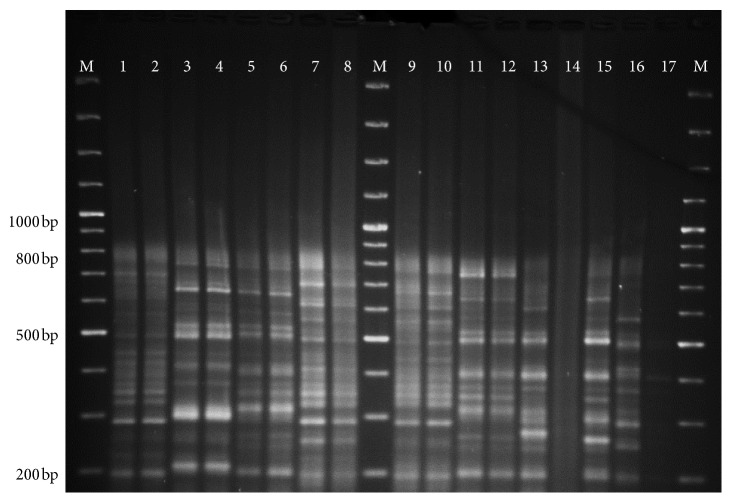
Gel picture of kDNA PCR-RFLP using HaeIII restriction digested products. Note: M: DNA ladder, Lane 1: BPK806, Lane 2: BPK813, Lane 3: BPK814, Lane 4: 63232-Fx, Lane 5: 63233-Fx, Lane 6: 63238-Fx, Lane 7: 63241-Fx, Lane 8: 63245-Fx, Lane 9: 63256-Fx, Lane 10: 63269-Fx, Lane 11: 63277-Fx, Lane 12: 63294-Fx, Lane 13: BPK-PKN435, Lane 14: BPK-PKN467, Lane 15: BPK-PKN468, Lane 16: BPK811, and Lane 17: BPK-PKN466.

**Figure 7:**
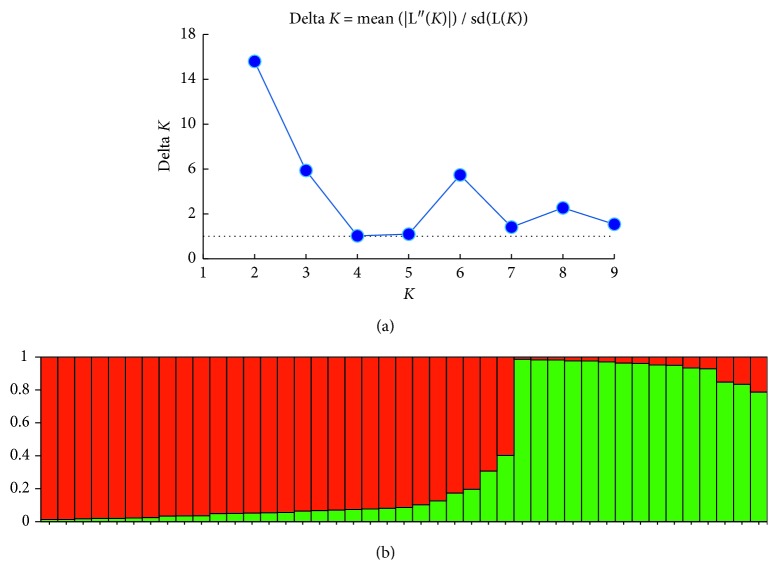
*L. donovani* kDNA genotypes determined by Structure v2.3.4. (a) Structure Harvester output for the determination of *K* (http://taylor0.biology.ucla.edu/structureHarvester/). Two distinct populations of kDNA genotypes are determined (*k* = 2). (b) Summary plot of estimates of *Q* value. Each individual data is represented by a single vertical line broken into *K* coloured segments, with length proportional to each of the *K* inferred clusters.

kDNA minicircle PCR-RFLP identified 18 different genotypes which were distributed as follows: six genotypes (K1, K2, K3, K4, K5, and K6) were isolated from hilly districts and remaining 12 genotypes (K7 to K18) were isolated from low-land districts as depicted in Figure 3. The interexperimental variability was controlled by testing the duplicate samples of parasite isolates in different gels and included in the dendrogram analysis as shown in Figure 4, and gel image of restriction fragments is shown in Figures 5 and 6. The distribution of kDNA genotypes is also depicted in Table 1. The output from the structure also showed the two distinct populations of kDNA (*K* = 2) as shown in Figure 7. Hence, two distinctly different *L. donovani* parasite populations were identified with kDNA genotyping.

Among two different approaches of genotyping, six kDNA genotypes (K1, K2, K3, K4, K5, and K6) were sharing same characteristics with group A *haspB* genotype. Other 12 kDNA genotypes (K7 to K18) had the same genetic characteristic with group B *haspB* genotype as shown in Figure 4.

## 4. Discussion

VL and PKDL are still considered as major public health problems in South Asian countries such as Nepal. PKDL cases are considered as reservoirs to maintain the endemicity during interepidemic period, but less attention has been given to explore the PKDL epidemiology. This may potentially jeopardize all the achievements of ongoing elimination programme. In fact, molecular epidemiology study has been limited to VL cases only [20, 21]. No such data are available in Nepalese PKDL cases. In this context, we aim to genetically characterize the *L*. *donovani* parasite causing VL and PKDL by the two assays (a) *haspB* PCR and (b) kDNA PCR-RFLP and explore the genetic similarity between these parasites with different clinical manifestations. This study differentiated the parasites into two different *haspB* genotypes, which is consistent with the finding of Bhattarai et al. We found that parasites isolated from hill districts had significantly different genotypes and PKDL were not identified in isolates from hill districts. The finding of this study indicates that the *L. donovani* genotypes have association with clinical features of leishmaniasis, and similar finding has also been demonstrated in Sri Lanka by Kariyaswami et al. [22]. This genotype diversity in these isolates needs further exploration to further confirm whether these clinical isolates are different, not only in their genetic makeup, but also in their clinical manifestation, epidemiology, antigenicity, and parasitic factor as well.

In addition, another molecular marker kDNA owing to its high amount of heterogeneity has been exploited by the molecular biologist for strain characterization in *Leishmania* to depict the microepidemiology of the parasite [20, 23, 24]. The kDNA RFLP is sensitive to interexperimental as well as interlaboratory variations, so robust protocol should be followed for reproducible result. To achieve reliable result, we used the standardized amount of DNA for PCR and RFLP assay, experimental control was included in each batch of RFLP, four consecutive subcultures of same isolates were studied, and for further robustness, all the samples were studied in duplicates. In this study, kDNA fingerprint showed the distinct clusters of two separate genotypes which were consistent with group A and group B, as determined by *haspB* PCR. This indicates further proof of principle that these isolates had genetically separate entity that influences the different clinical manifestations such as visceral or dermal manifestation in patients. Similar findings have been reported from the Indian subcontinent using *β-*tubulin gene as a probe where parasites from VL and PKDL were randomly clustered in between different assigned genotypes [9]. Another study using probe Ldp13 also showed genetic heterogeneity among VL and PKDL isolates from the Indian subcontinent [10]. This is particularly interesting because the Sudanese isolates were found to have similar genetic makeup by various PCR fingerprinting methods [25]. The mechanism of parasite diversification between VL and PKDL is still a matter of research, but some evidence points towards the contributory role of immune response of patient [2].

Ostyn et al. demonstrated that the VL infections at hilly districts were locally transmitted, and in this study, we also found the different *L. donovani* genotypes circulating in hilly districts. Hence, Ostyn et al. documented the expansion of VL in new endemic hilly areas of Nepal which is verified by the evidence obtained from the study of epidemiology, microbiology, clinical, and entomology [16]. Moreover, *Phlebotomus argentipes* sandfly infected with *L. donovani* were identified, and hence, the asymptomatic infections were also reported in hilly districts by Ostyn et al. This might be the influence of recent increasing trends of temperature in hills due to the global warming that favours the breeding of sandfly vector populations. Therefore, it is an urgent need to explore the role of PKDL as a probable reservoir for VL in order to understand the transmission dynamic of VL together with global impact of climate change.

## 5. Conclusions

This study concludes that PKDL and VL clinical parasite isolates were genetically separated and most of them confined to particular geographic location of Nepal. However, different molecular tools are developed elsewhere [26]; the high discriminatory power of kDNA PCR-RFLP tool could be useful in the analysis of molecular epidemiology of VL and PKDL in future.

## Figures and Tables

**Table 1:** Genotypes of clinical isolates and their geographical origin.

VL/PKDL	Isolates	Origins		Genotypes	
		District	VDC	*haspB*	kDNA RFLP
VL	BPK806/0	Bhojpur	Pawala	A	K4
VL	BPK811/0	Saptari	Rajbiraj	B	K16
VL	BPK813/0	Morang	Biratnagar	B	K10
VL	BPK814/0	Jhapa	Chandragadhi	B	K9
VL	63232-Fx	Bhojpur	Mane Bhanjyang	A	K1
VL	63233-Fx	Bhojpur	Jarayotar	A	K2
VL	63238-Fx	Jhapa	Gauradaha	B	K8
VL	63241-Fx	Jhapa	Maheshpur	B	K12
VL	63245-Fx	Morang	Biratnagar	B	K11
VL	63256-Fx	Morang	Dewanganj	B	K13
VL	63269-Fx	Khotang	Wopung	A	K6
VL	63277-Fx	Sunsari	Dharan	A	K3
VL	63291-Fx	Sunsari	Chatara	B	K14
VL	63294-Fx	Okhaldhunga	Obu	A	K5
PKDL	BPK-PKN435	Morang	Jahada	B	K18
PKDL	BPK-PKN466	Morang	Majhare	B	K15
PKDL	BPK-PKN467	Siraha	Siraha	B	K17
PKDL	BPK-PKN468	Saptari	Rupnagar	B	K7

## Data Availability

This is a hospital-based study. Samples were collected during the routine diagnostic and genotyping procedure. Therefore, the data of the analysis are available upon request from the corresponding author or Head, Department of Microbiology (hod.microbiology@bpkihs.edu), B. P. Koirala Institute of Health Sciences, Dharan, Nepal.
